# Supplementary material for: Integrated Multi-Omics Analysis Uncovers Immune–Metabolic Interplay in Hepatocellular Carcinoma Tumor Microenvironment
Source: Cancers (Basel). 2025 Nov 3;17(21):3565. doi: 10.3390/cancers17213565 (PMC12610199; doi:10.3390/cancers17213565)
Supplement: Supplementary file 1 [file cancers-17-03565-s001.zip › cancers-3915783-supplementary.pdf]

Supplementary Material

Integrated Multi-omics Analysis Uncovers Immune-Metabolic Interplay in Hepatocellular Carcinoma Tumor Microenvironment

Jong-Heon Park, Dae Won Sim, Sook-Young Kim, Joon Young Choi, Seung Hyup Hyun*, Je-Gun Joung*

* Co-corresponding authors

Supplementary Methods

Table S1. The clinical characteristics of the three groups, including their effect sizes

Table S2. Significance and effect sizes of differences in cytolytic activity (CYT) score, ESTIMATE score, gene expression profile (GEP) score, and immune score

Table S3. Enriched pathways in Inflamed and non-Inflamed HCC groups

Figure S1. Comparison of tumor mutation burden (TMB) among cluster groups

Figure S2. Heatmap of differentially expressed genes in inflamed and non-inflamed HCC groups

Figure S3. Dotplot showing the expression of marker genes in scRNA-seq dataset

Supplementary Methods

Immune cell-type deconvolution methods

EPIC (Estimating the Proportions of Immune and Cancer Cells): *EPIC* incorporates RNA-seq-based reference gene expression profiles of nonmalignant and immune cells [12]. The *EPIC* R package was used to estimate cell-type proportions from $\log_2$(TPM) input data. Analyzed cell types included *B cells*, *CD4+ T cells*, *CD8+ T cells*, *macrophages*, and *NK cells*. *EPIC* is available as a web tool (http://epic.gfeller-lab.org) and an R package (https://github.com/GfellerLab/EPIC).

xCell 2.0: *xCell 2.0* estimates cell-type proportions in tissues from bulk RNA-seq data [13]. The *xCell* R package was applied to $\log_2$(TPM) data. Analyzed cell types included *aDC*, *B cells*, *basophils*, *CD4+ memory T cells*, *CD4+ Tcm*, *CD8+ T cells*, *CD8+ Tcm*, *cDC*, *DC*, *eosinophils*, *iDC*, *M1 macro-phages*, *M2 macrophages*, *mast cells, monocyte, neutrophils, NKT, plasma cells, Th1 cells, and Th2 cells. xCell* is available as an R package (https://github.com/dviraran/xCell).

MCP-counter (Microenvironment Cell Populations-counter): *MCP-counter* quantifies immune and stromal cell abundances from bulk RNA-seq data (https://github.com/ebecht/MCPcounter). The *MCPcounter* R package was used with counts per million (CPM) as input. Analyzed cell types included *T cells*, *CD8+ T cells*, *cytotoxic lympho-cytes*, *NK cells*, *myeloid dendritic cells*,

and *neutrophils*.

quanTIseq (Quantification of the Tumor Immune Contexture from Human RNA-seq Data): *quanTIseq* quantifies immune cell proportions in the tumor micro-environment from bulk RNA-seq data. The *quantiseqr* R package was used with Transcripts Per Million (TPM) input. Analyzed cell types included *B cells*, *M1 macrophages*, *M2 macrophages*, *monocytes*, *CD4+ T cells (non-regulatory)*, *CD8+ T cells*, *Tregs*, and *myeloid dendritic cells*. *quanTIseq* is available via a web interface (http://icbi.at/quantiseq).

Table S1. The clinical characteristics of the three groups, including their effect sizes.

Characteristics	Overall (n = 60)	Cluster 1 (n = 7)	Cluster 2 (n = 27)	Cluster 3 (n = 26)	p-value	effect size *r*
**Age (range, years)**	58.1(42-76)	51.0(42-61)	60.0(44-76)	57.0(43-73)	0.0629	0.060
**Sex**					0.6951	
Male	49 (81.7%)	5 (71.4%)	23 (85.1%)	21 (80.7%)		0.1101
Female	11 (18.3%)	2 (28.6%)	4 (14.9%)	5 (19.3%)		
**Tumor size(cm)**	4.4 (±1.4)	5.3 (±1.5)	4.0 (±2.0)	4.5 (±2.0)	0.0767	0.050
**Tumor SUV**[1]	4.5 (±2.6)	8.9 (±5.0)	3.7 (±1.1)	4.2 (±1.9)	0.023	0.1179
**Etiology**					0.5355	
HBV	46 (76.7%)	6 (85.7%)	22 (81.4%)	18 (69.2%)		0.1616
HCV	5 (8.3%)	1 (14.3%)	2 (7.4%)	2 (7.7%)		
Alcohol and others	9 (15%)	0 (0.0%)	3 (11.2%)	6 (23.1%)		
**Cirrhosis**					0.5923	
Yes	22 (36.7%)	3 (42.8%)	8 (29.6%)	11 (42.3%)		0.1321
No	38 (63.3%)	4 (57.2%)	19 (70.4%)	15 (57.7%)		
**Microvessel invasion**					0.0275	
Yes	30 (50.0%)	6 (85.7%)	9 (33.3%)	15 (57.7%)		0.3461
No	30 (50.0%)	1 (14.3%)	18 (66.7%)	11 (42.3%)		

Table S2. Significance and effect sizes of differences in cytolytic activity (CYT) score, ESTIMATE score, gene expression profile (GEP) score, and immune score.

	p-value	η^2	ω^2
Immune Score	9.75E-15	0.6776053	0.6625458
ESTIMATE Score	1.18E-12	0.6185045	0.6010956
CYT Score	1.33E-05	0.3255396	0.2983441
GEP Score	3.66E-10	0.5334726	0.5129052

Table S3. Enriched pathways in Inflamed and non-Inflamed HCC groups.

**Enrichment pathway analysis of inflamed group genes**				
No	Name	p-value	padj	Odss ratio
1	Immune System	5.E-06	4.E-04	7.15

2	Innate Immune System	5.E-04	2.E-02	6.08
3	RHO GTPase Cycle	3.E-03	5.E-02	7.96
4	Cytokine Signaling in Immune System	3.E-03	5.E-02	5.93
5	Neutrophil Degranulation	3.E-03	5.E-02	7.48
6	Interferon Gamma Signaling	7.E-03	9.E-02	17.08
	Enrichment pathway analysis of non-inflamed group genes			
1	Metabolism	7.E-13	9.E-11	10.42
2	Bile Acid and Bile Salt Metabolism	2.E-07	1.E-04	51.07
3	Recycling of Bile Acids and Salts	7.E-06	2.E-04	99.71
4	Biological Oxidations	8.E-06	2.E-04	14.82
5	Metabolism of Amino Acids and Derivatives	1.E-05	3.E-04	10.86
6	Synthesis of Bile Acids and Bile Salts via 7Alpha-Hydroxycholesterol	2.E-05	3.E-04	74.76
7	Intrinsic Pathway of Fibrin Clot Formation	2.E-05	3.E-04	74.76
8	Fructose Catabolism	4.E-05	7.E-04	324.46
9	Synthesis of Bile Acids and Bile Salts	5.E-05	7.E-04	49.82
10	Choline Catablism	7.E-05	8.E-04	243.33

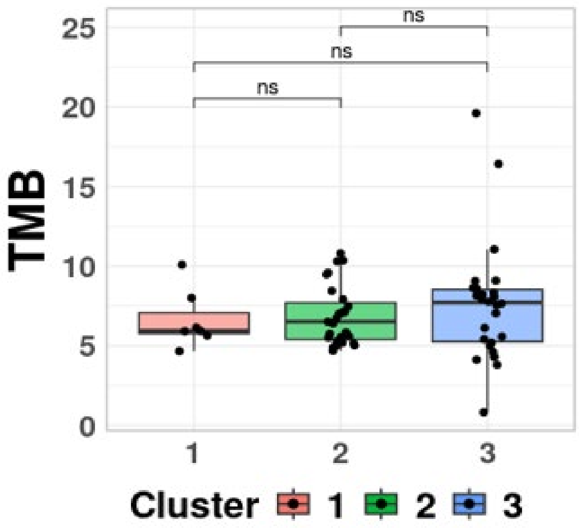

Figure S1. Comparison of tumor mutation burden (TMB) among cluster groups.

(a) (b)

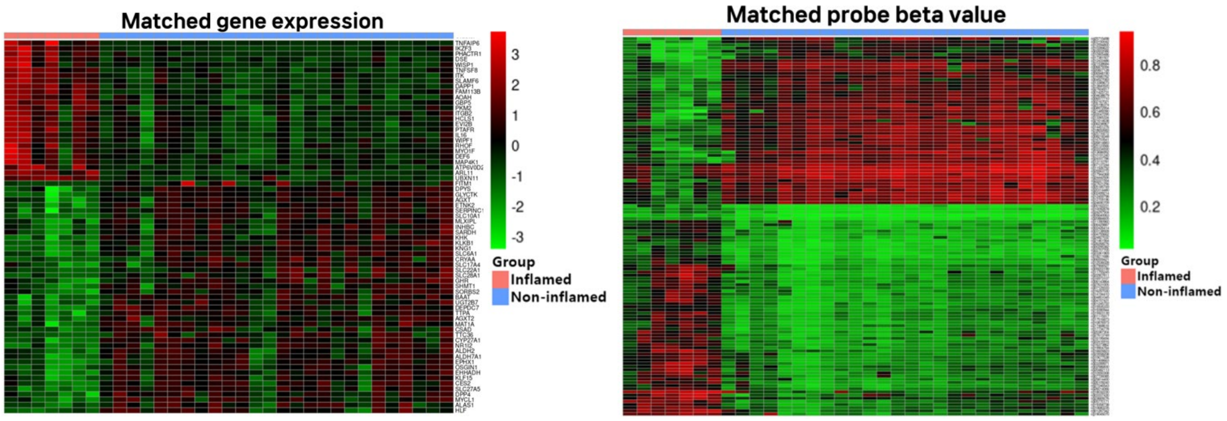

Figure S2. Heatmap of differentially expressed genes in inflamed and non-inflamed HCC groups. (a) Differential gene expression profile between groups. (b) Beta values of methylation probes located in differentially expressed genes.

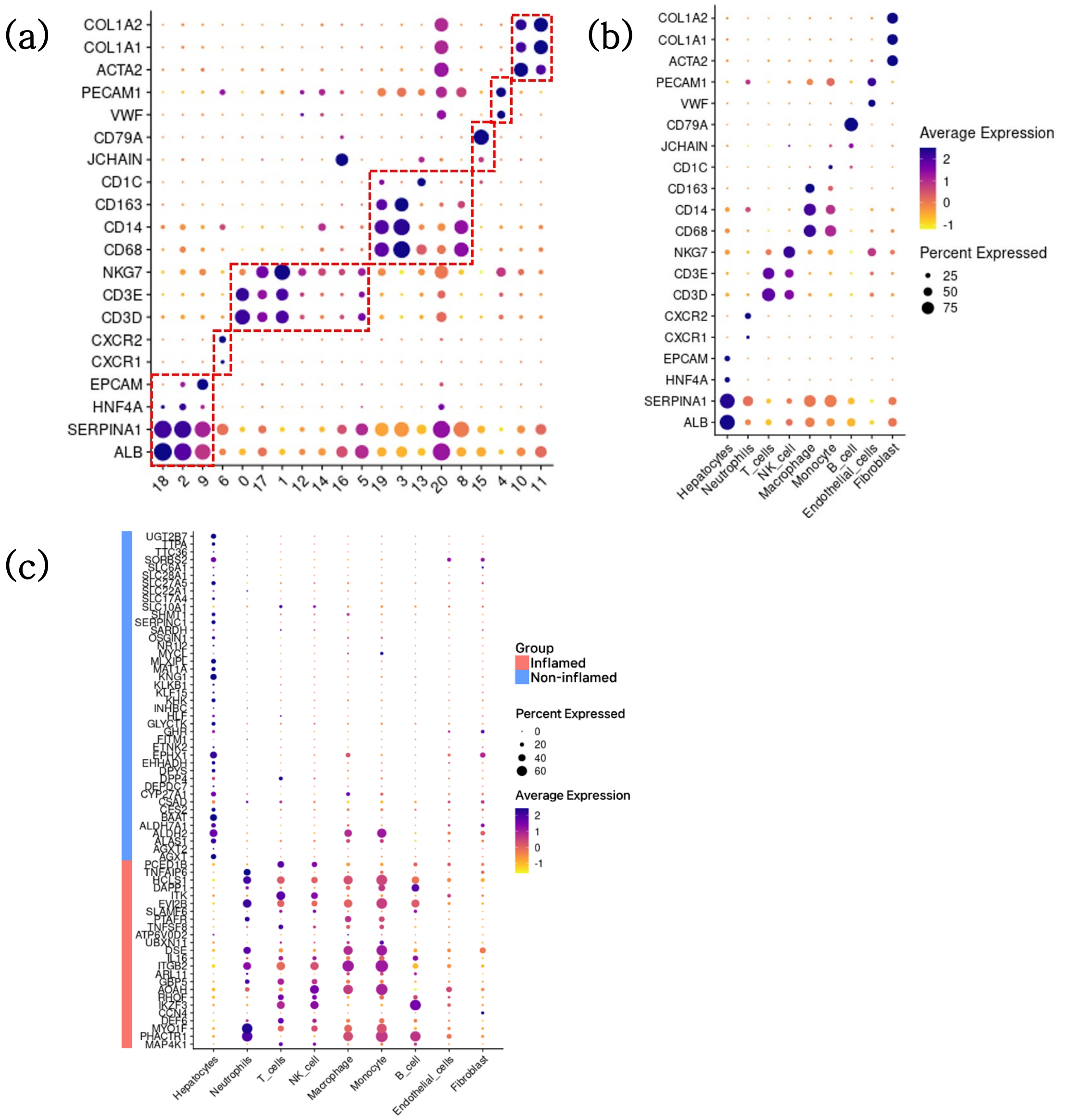

Figure S3. Dotplot showing the expression of marker genes in scRNA-seq dataset. (a) Expression of known tissue-specific marker genes in clusters. (b) Expression of known tissue-specific marker genes in cell-types. (c) Expression of marker genes associated with the inflamed and non-inflamed HCC groups across cell types.
